# Veno-arterial extracorporeal membrane oxygenation in a patient with septic cardiomyopathy induced by severe community-acquired pneumonia due to *Acinetobacter baumannii*: A case report

**DOI:** 10.1097/MD.0000000000042092

**Published:** 2025-05-23

**Authors:** Yan-Na Jiao, Jian-Biao Meng

**Affiliations:** a Department of Critical Care Medicine, The First Affiliated Hospital, Zhejiang University School of Medicine, Hangzhou, Zhejiang Province, PR China; b Department of Critical Care Medicine, Tongde Hospital of Zhejiang Province, Hangzhou, Zhejiang Province, PR China.

**Keywords:** AB, cardiogenic shock, septic cardiomyopathy, severe community-acquired pneumonia, VA-ECMO

## Abstract

**Rationale:**

Community-acquired pneumonia due to *Acinetobacter baumannii* (CAP-AB) is uncommon; however, its mortality is extremely high because of severe pneumonia, septic shock, and multiple organ dysfunction syndrome including septic cardiomyopathy and cardiogenic shock. Veno-arterial extracorporeal membrane oxygenation (VA-ECMO), an important component of treatment in the early stage of septic cardiomyopathy can affect the prognosis of similar patients.

**Patient concerns:**

A 65-year-old man presented to the fever clinic with fever, cough, and stuffiness for 1 day. On admission, he manifested hypoxemia and hypotension, and chest computed tomography showed pneumonia, and *Acinetobacter baumannii* (AB) was positive in bronchoalveolar lavage fluid tested by metagenomic next-generation sequencing (mNGS).

**Diagnoses:**

Community-acquired pneumonia (CAP), respiratory failure, septic shock, septic cardiomyopathy, and cardiogenic shock.

**Interventions:**

As the diagnosis of septic shock, septic cardiomyopathy and cardiogenic shock induced by CAP-AB and respiratory failure were made, cefoperazone/sulbactam 3 g q8h, moxifloxacin 400 mg qd, inotropes and vasopressors and mechanical ventilation were initiated. However, although global end diastolic volume index was 744 mL/m^2^, hypotension and tachycardia remained, the left ventricular ejection fraction was 30%, and circulatory failure (cardiogenic shock) did not improve. Hence, VA-ECMO was applied to assist circulation on the day of admission due to the involvement of septic cardiomyopathy and cardiogenic shock.

**Outcomes:**

On day 2, tachycardia improved, left ventricular ejection fraction increased to 54%, and VA-ECMO was withdrawn on day 5. On day 10, mechanical ventilation was withdrawn and the tracheal cannula was removed. Subsequently, the patient was transferred to the respiratory department on day 14.

**Lessons:**

A patient with septic cardiomyopathy and cardiogenic shock induced by severe CAP-AB was treated with VA-ECMO in the early stage. Patients with CAP-induced septic cardiomyopathy may benefit from the introduction of VA-ECMO during the early stage. Further studies are required to evaluate the advantages and disadvantages of early VA-ECMO in patients with CAP-induced septic cardiomyopathy.

## 1. Introduction

Community-acquired pneumonia due to *Acinetobacter baumannii* (CAP-AB) is uncommon in the community; however, its mortality is as high as 64% because of severe pneumonia, septic shock, and multiple organ dysfunction syndrome (MODS).^[1,2]^ Septic cardiomyopathy is a severe complication of septic shock and contributes to poor prognosis and high mortality in patients with septic shock.^[3]^ Veno-arterial extracorporeal membrane oxygenation (VA-ECMO), a device of extracorporeal life support, has been widely used for severe cardiogenic shock. Asaki et al reported that VA-ECMO might be effectiveness in a patient with circulatory insufficiency due to septic shock on day 3 since admission.^[4]^ We report the successful and effective treatment in a patient with severe CAP-AB induced septic cardiomyopathy by introduction of VA-ECMO in the early stage of septic cardiomyopathy.

## 2. Case presentation

A previously healthy 65-year-old man presented to the fever clinic with fever, cough, and stuffiness for 1 day. Upon arrival at the intensive care unit (ICU), the patient manifested critical illness with unstable vital signals: body temperature, 39.3°C; heart rate, 142 beats/min with regular heart rhythm; respiratory rate, 38 breaths/min; blood pressure, 87/42 mm Hg; and pulse oxygen saturation was between 86% and 90% when the fraction of inspired oxygen (FiO_2_) was 0.6. Dry oral mucosa and coarse crackles were observed in the left lung (reference ranges of physiological indexes, see Table S1, Supplemental Digital Content, https://links.lww.com/MD/P15).

**Figure 1.**
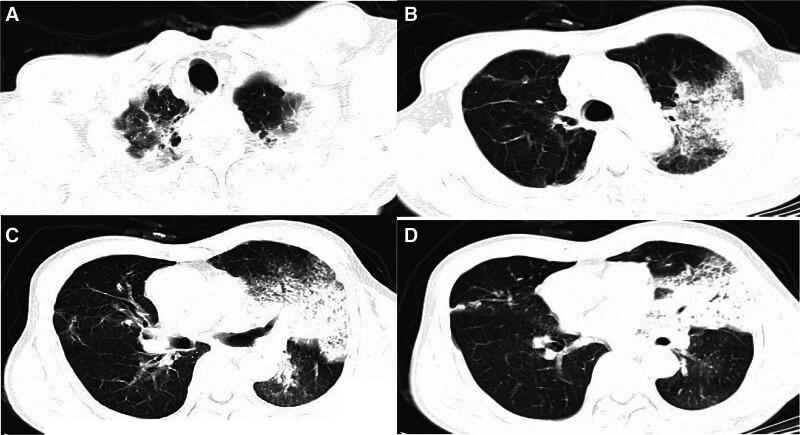
Computed tomography obtained in the fever clinic shown severe infiltrate and consolidation in the left upper lung field (A–D) and mild infiltrate in the right apex pulmonis (A) and left lower lung field (C and D).

The blood examination values were as follows: white blood cell count, 2600/mL; hemoglobin 156 g/L; platelet count 127,000/mL, and high-sensitivity C-reactive protein 335 mg/L. The arterial blood gas analysis on FiO_2_ of 0.6, pH 7.425, PaO_2_ 60 mm Hg, PaO_2_/FiO_2_ 100 mm Hg, HCO_3_^‐^ 15.4 mmol/L, and lactic acid of 8.1 mmol/L. High-sensitivity troponin T, heart type-fatty acid binding protein, and N-terminal pro B-type natriuretic peptide levels were 0.018 ng/L, 19.7 ng/mL, and 8679 pg/mL, respectively, and procalcitonin was above 100 ng/mL. Blood samples were also cultured. Due to severe hypoxemia, intubation and mechanical ventilation were administered when the patient was admitted to ICU. The sputum samples for culture and a sample of bronchoalveolar lavage fluid (BALF) tested by the metagenomics next-generation sequencing (mNGS) were collected after intubation. Chest computed tomography obtained in fever clinic revealed severe infiltration and consolidation in the left upper lung field and mild infiltration in the right apex pulmonis and left lower lung field (Fig. 1). Transthoracic echocardiogram initially showed obviously reduced constriction function of the left ventricle and left ventricular ejection fraction (LVEF) of 30%, while tricuspid annular plane systolic excursion was 12 mm, right ventricular global longitudinal strain: absolute value was 15%. After admission, an arterial line catheter, central line catheter, and pulse indicator continuous cardiac output (PiCCO) catheter were promptly inserted. The initial invasive hemodynamic status on the PiCCO device showed a central venous pressure of 25 mm Hg, arterial blood pressure of 89/61 mm Hg (norepinephrine at 0.57 µg/kg/min and dobutamine at 5.11 µg/kg/min), global end diastolic volume index of 744 mL/m^2^, cardiac index of 1.59 mL/m^2^/min, systemic vascular resistance index of 2214 dyns/cm^5^m^2^, and extravascular lung water index of 11 mL/kg. The initial acute physiology and chronic health evaluation-II and sequential organ failure assessment scores were 35 and 13, respectively. Therefore, we made the presumptive diagnosis of severe community-acquired pneumonia (CAP), respiratory failure (I type), septic shock, septic cardiomyopathy, and acute kidney injury (reference ranges of physiological indexes, see Table S1, Supplemental Digital Content, https://links.lww.com/MD/P15 and reference ranges of laboratory indices, see Table S2, Supplemental Digital Content, https://links.lww.com/MD/P15).

**Figure 2.**
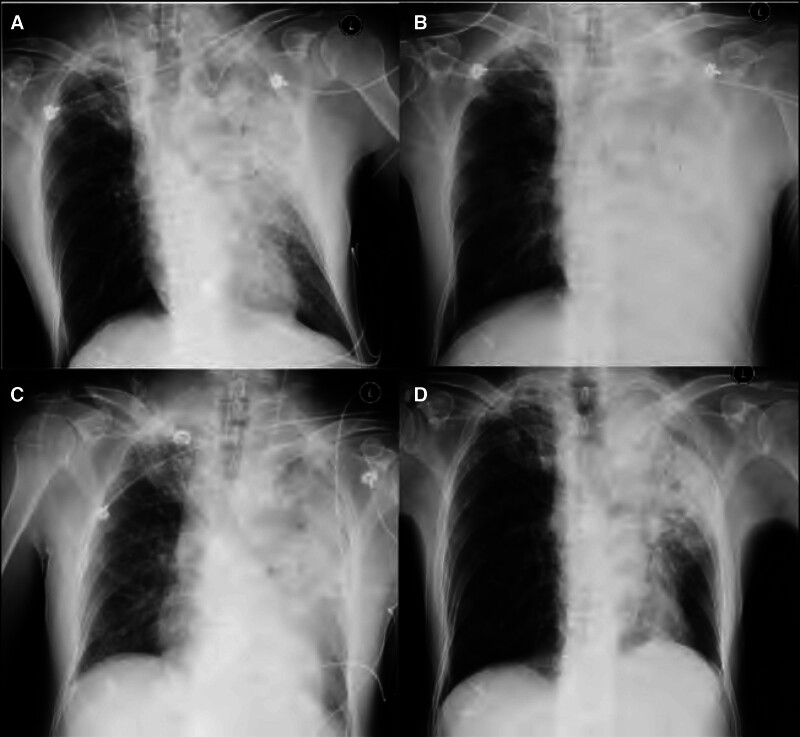
Simple radiographs during the course of VA-ECMO shown the progress of the lung infiltrates, consolidation and pleural effusion. (A) A simple radiograph on arrival at ICU shown severe infiltrate and consolidation in the left upper lung field and mild infiltrate in the right apex pulmonis and left lower lung field. (B) A simple radiograph on day 2 shown severe infiltrate and consolidation in the left lung field and moderate infiltrate in the right apex pulmonis field and left pleural effusion. (C) A simple radiograph on day 5 shown severe infiltrate and consolidation in the left lung field, less severe infiltrate and consolidation in the left lower lung field and reduced left pleural effusion. (D) A simple radiograph on day 30 shown partial consolidation and fibration was on left upper lung. ICU = intensive care unit, VA-ECMO = veno-arterial extracorporeal membrane oxygenation.

Cefoperazone/sulbactam 3.0 Q8h and moxifloxacin 400 mg QD were administered intravenously during the first hour of treatment. fluid resuscitation and noradrenaline at 0.57 µg/kg/min and dobutamine at 5.11 µg/kg/min were initiated to correct hypotension and improve tissue perfusion. His condition did not improve, but was aggravated in 2 hours. Continuous venous infusion noradrenaline at 1.03 µg/kg/min and adrenaline at 0.29 µg/kg/min to keep mean arterial pressure (MAP) being above 65 mm Hg. Transthoracic echocardiogram revealed dilation of the left ventricle and LVEF of approximately 20%. The N-terminal pro B-type natriuretic peptide and high-sensitivity troponin T levels increased to 10,662 pg/mL and 0.021 ng/L, respectively. The patient was already in cardiogenic shock at the time of the initial PiCCO measurement. Arterial blood gas analysis was pH of 7.25, PaO_2_ of 134 mm Hg, PaO_2_/FiO_2_ of 134 mm Hg, HCO_3_^‐^ of 14.5 mmol/L, and lactic acid of 8.1 mmol/L when the ventilator mode of pressure control ventilation with FiO_2_ of 1.0, RR of 15 breaths/min, pressure of control of 16 cmH_2_O and positive end expiratory pressure of 10 cmH_2_O. Aggressive fluid resuscitation with 30 mL/kg crystalloids achieved a cumulative positive balance of +3.5 L within the first 6 hours. After ultrasound-guided percutaneous femoral access (Maquet 23Fr venous/17Fr arterial cannulae) and ACT-guided heparinization (target 180–220 seconds). Percutaneous femoral VA-ECMO was implanted under ultrasound guidance, achieving a flow rate of 3.5 L/min (60 mL/kg/min), adrenaline was discontinued, and the noradrenaline dosage was reduced to 0.28 µg/kg/min. In this condition, the HR was no more than 100 beats/min, as the MAP was above 65 mm Hg, central venous pressure decreased from 25 to 16 mm Hg after VA-ECMO. However, on day 2, the simple radiograph still showed severe infiltration and consolidation in the left lung field and moderate infiltration in the right apex pulmonis field and left pleural effusion compared to on arrival at the ICU (Fig. 2A, B). In the first 72 hours after admission to ICU, the input volume and output volume were 12,500 mL and 10,700 mL, respectively, and the fluid balance was +1800 mL, which was based on furosemide 20 mg iv q6h in the first 72 hours. On day 5, because nordrenaline was discontinued, arterial blood gas analysis was pH of 7.47, PaO_2_ of 99.7 mm Hg, PaO_2_/FiO_2_ of 249 mm Hg, HCO_3_^‐^ of 23.4 mmol/L, and lactic acid of 1.9 mmol/L when the ventilator mode of pressure control ventilation with FiO_2_ of 0.4, RR of 15 breaths/min, pressure of control of 15 cmH_2_O and positive end expiratory pressure of 6 cmH_2_O, LVEF improved to 54%, tricuspid annular plane systolic excursion was 18 mm, right ventricular global longitudinal strain: absolute value was 24% and less severe infiltrate and consolidation in the left lower lung field and reduced left pleural effusion on a simple radiograph (Fig. 2B, C), VA-ECMO was withdrawn. Mechanical ventilation was discontinued, and the tracheal cannula was removed on day 10. During VA-ECMO, jaundice, bleeding, and other common complications did not develop. Subsequently, the patient was transferred to the respiratory department on day 14. On day 30, simple radiography showed that the left lower lung consolidation was absorbed, but partial consolidation and fibration were observed in the left upper lung (Fig. 2D) (reference ranges of physiological indexes, see Table S1, Supplemental Digital Content, https://links.lww.com/MD/P15 and reference ranges of laboratory indices, see Table S2, Supplemental Digital Content, https://links.lww.com/MD/P15).

**Figure 3.**
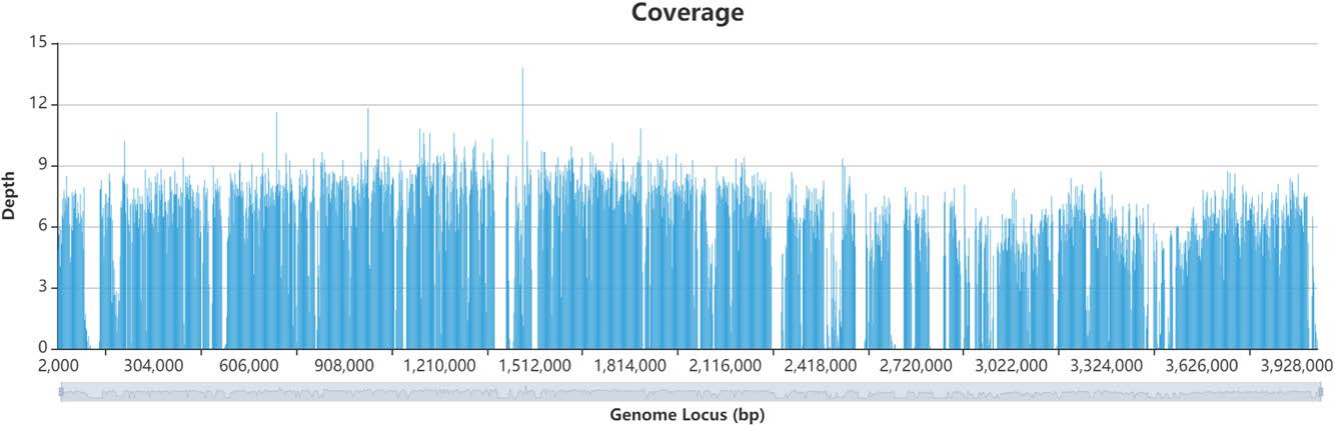
Coverage of AB genome locus (bp). AB: *Acinetobacter baumannii.*

As for the pathogen, on day 2, the mNGS result of BALF showed *Acinetobacter baumannii* (AB) with number of classified sequence reads of 40,638 and relative abundance of 71.97% relative abundance. The coverage of the AB genome locus (bp) is shown in Figure 3. On day 3, the culture of blood and sputum samples indicated AB, which was isolated from both blood and sputum samples, and its sensitivity to antibiotics was measured. The isolated AB was susceptible to cefoperazone/sulbactam, ceftazidime, piperacillin/tazobactam, cefepime, imipenem, amicacin, levofloxacin, tigecycline, and sulfamethoxazole and moderately tolerant to ceftriaxone (Table 1).

**Table 1:** The antibiotic sensitivity test of AB.

No	Antimicrobial	Sputum culture		Blood culture	
		Interpretation*	MIC, µg/mL	Interpretation*	MIC, µg/mL
1	Piperacillin/tazobactam	S	8	S	8
2	Ceftazidime	S	8	S	8
3	Ceftriaxone	I	16	I	16
4	Cefoperazone/sulbactam	S	≤8	S	≤8
5	Cefepime	S	8	S	8
6	Imipenem	S	≤0.25	S	≤0.25
7	Amicacin	S	≤2	S	≤2
8	Levofloxacin	S	2	S	2
9	Tigecycline	S	≤0.5	S	≤0.5
10	Compound sulfamethoxazole	S	≤20	S	≤20

AB = *Acinetobacter baumannii*, MIC = minimum inhibitory concentration.

* I = intermediate, S = susceptible.

## 3. Discussion

This case report describes a 65-year-old man with septic cardiomyopathy induced by severe community-acquired pneumonia due to AB who was treated with early introduction of VA-ECMO. mNGS of BALF confirmed AB, and the antibiotic susceptibility profile of AB in blood culture was the same as that in sputum culture.

CAP is defined as infection acquired outside of the hospital setting.^[5,6]^ CAP-AB is rather rare, but it is extremely severe in the world, especially in the tropical regions. Compared to hospital-acquired pneumonia due to AB (HAP-AB), CAP-AB is characterized by fulminant pneumonia with or without bloodstream infection and manifests acute onset of respiratory symptoms followed by septic shock and MODS.^[7]^ The patient in this report suffered from septic shock induced by severe CAP-AB, then progressed to MODS, including septic cardiomyopathy, which is consistent with the manifestations of severe CAP-AB shown in *the Expert Review* by Dexter C et al.^[1]^ Although epidemiologic studies indicate alcoholism is associated with severe CAP-AB,^[1]^ he is not interested in alcohol. Moreover, the patient was previously healthy without other risk factors of CAP-AB, such as smoking, diabetes mellitus, renal disease, and structural lung disease. Because the risk factors for developing severe CAP-AB in this patient are still unknown, we suppose that severe CAP-AB could develop in a healthy person without the risk factors mentioned above.

In the patient, we hypothesized that lower LVEF is associated with cardiac dysfunction induced by sepsis (septic cardiomyopathy). The incidence of septic cardiomyopathy in the ICU ranges between 10% and 70%.^[3]^ Septic cardiomyopathy is often related to increased cytokines and components of the complement cascade due to lipopolysaccharide.^[8]^ Lower LVEF and elevated cardiac troponins in sepsis facilitate the diagnosis of septic cardiomyopathy.^[8–10]^

Reports on the use of VA-ECMO in patients with septic cardiomyopathy are rare. By reviewing the literature, we found that Asaki et al described VA-ECMO was used to treat a patient with septic cardiomyopathy due to *Legionella* pneumonia in 2018.^[4]^ Serota et al reported that they treated severe CAP-AB without septic cardiomyopathy using VV-ECMO.^[11]^ However, to date the use of VA-ECMO in patients with septic cardiomyopathy induced by severe CAP-AB has not been reported. In this case, VA-ECMO was not only introduced for patients with septic cardiomyopathy due to severe CAP-AB but was also initiated in the early stage of septic cardiomyopathy. However, the timing of VA-ECMO initiation in patients with septic cardiomyopathy remains unclear. In the case reported by Asaki et al, VA-ECMO was introduced on day 3 in the ICU,^[4]^ but the patient in our ICU was treated with VA-ECMO on day 1 when he was admitted. Although the timing of initiation of VA-EMCO in the 2 patients was different, both patients experienced severe CAP, followed by septic cardiomyopathy (LVEF ≤ 20%) and even cardiogenic shock when VA-ECMO was introduced. The reasons for the difference in the timing of VA-ECMO introduction in the 2 patients are as follows. First, 2 patients were diagnosed with severe CAP and septic shock, but the causative pathogens of CAP were *Legionella* and AB, respectively. Second, the incidence of CAP-AB is less than that of *Legionella* CAP; therefore, administration of empirical antibiotics to CAP patients sometimes does not cover AB. Finally, compared with *Legionella* pneumonia, AB pneumonia is more rapid and fulminant in the first 36 hours and its higher mortality has reached 64%.^[1]^ In Korea, a 53-year-old male patient with fulminant CPA-AB within 36 hours after admission.^[12]^

Furthermore, we believe that early VA-ECMO can improve the prognosis of patients with septic cardiomyopathy induced by severe CAP when sepsis-induced MODS or septic cardiomyopathy starts.^[13]^ Early introduction of VA-ECMO can enhance tissue perfusion and oxygenation as soon as possible by contributing to intake of oxygen and removal of carbon dioxide and maintaining stable MAP.^[14]^ The 2 patients with severe CAP due to *Legionella* and AB all benefited from early VA-ECMO as they were survivors. On the contrary, the patient with severe CAP-AB reported by Oh et al expired in 36 hours after admission without early VA-ECMO.^[12]^

There are some therapeutic rationale of VA-ECMO in sepsis-induced cardiomyopathy and cardiogenic shock early.^[15]^ First, hemodynamic and respiratory stabilization: cardiac augmentation: VA-ECMO assumes partial/full cardiopulmonary bypass function, effectively unloading ventricular workload.^[16]^ Second, oxygenation optimization: the integrated membrane oxygenator ensures adequate gas exchange, correcting tissue hypoxia secondary to cardiogenic pulmonary edema or low-flow states.^[17]^ Third, end-organ perfusion maintenance: in sepsis-related myocardial dysfunction (LVEF < 35%) and shock states (MAP < 65 mm Hg), VA-ECMO sustains end-organ perfusion pressure, mitigates progressive MODS manifestations, demonstrates 23% reduction in sequential organ failure assessment score increments within 72 h.

While VA-ECMO has been demonstrated substantial therapeutic value sepsis-induced cardiomyopathy and cardiogenic shock, its application necessitates rigorous risk stratification and mitigation of the following complications.^[18]^ First, hemorrhagic/thrombotic diathesis, the requisite anticoagulation (ACT 160–180 seconds) and foreign surface exposure predispose to both hemorrhagic diathesis (particularly intracranial and surgical site bleeding) and circuit-associated thrombosis, occasionally progressing to disseminated intravascular coagulation. Second, catheter-related bloodstream infections, prolonged ECMO cannulation elevates catheter-related bloodstream infections risk, mandating strict aseptic protocols and early escalation to culture-directed antimicrobial prophylaxis when febrile episodes occur with elevated procalcitonin (>2 ng/mL). Third, distal limb ischemia, femoral arterial cannulation carries 18% to 22% incidence of limb hypoperfusion, necessitating distal perfusion catheter placement (8–10Fr) with continuous near-infrared spectroscopy monitoring (threshold <40% saturation for >30 minutes indicating intervention). Last, mechanical hemolysis, high-flow turbulence induces subclinical hemolysis (plasma free hemoglobin >50 mg/dL), requiring daily haptoglobin assessment and circuit inspection for thrombus formation when lactate dehydrogenase exceeds 800 U/L.

To understand the pathogen of severe CAP as soon as possible, we collected the BALF when intubation was completed and sent BALF to the Medical Center of Precision Diagnosis in our hospital for mNGS testing. The result of the mNGS test for BALF was AB and was reported in <24 hours. Sputum and blood samples were cultured simultaneously. The results of sputum and blood sample cultures were AB in sputum and blood, respectively, and blood samples had the same sensitivity to antibiotics. In this case, we found that we could obtain positive results of sputum and blood sample cultures in almost 2 days, but it still took more time to culture bacteria than mNGS for BALF, so we believe that mNGS is a more effective, timely, and precise method for recognizing pathogens.^[19,20]^ Combining mNGS with bacterial culture and sensitivity in this case, we not only understood the pathogen of severe CAP in a timely manner, but also knew the sensitivity to antibiotics precisely, which could guide the use of antibiotics. According to the results of bacterial culture and sensitivity in this case, cefoperazone/sulbactam was not discontinued until the patient was transferred to the ward of the respiratory department.

mNGS demonstrates significant clinical utility through its capacity for unbiased pan-pathogen detection, encompassing bacteria, viruses, fungi, and antimicrobial resistance genes.^[21]^ This technology overcomes the constraints of conventional culture methods by achieving 1–10 genomic copies/μL sensitivity and reducing diagnostic turnaround time by 3- to 5-fold (24–48 hours). Notably, its 85% to 92% concordance between resistance gene profiles and phenotypic susceptibility testing enables targeted antimicrobial therapy, aligning with precision medicine principles.^[21]^ But it has the core limitations. Operational challenges include high costs and technical complexity requiring specialized bioinformatics pipelines. Diagnostic accuracy may be compromised by false positives from environmental/host DNA interference and missed low-abundance pathogens (>0.1% total DNA detection threshold).^[22]^ Furthermore, clinical implementation necessitates multimodal correlation-integrating host inflammatory biomarkers (procalcitonin/CRP) and imaging evidence to distinguish colonization from active infection, adding interpretive complexity.

Clinical performance metrics reveal 92% to 98% sensitivity for cerebrospinal fluid/tissue specimens (vs 60%–75% with culture) and 65% to 80% sensitivity for blood samples (specificity >90%).^[23]^ The IDSA guidelines endorse prioritized use in immunocompromised hosts and central nervous system infections, where mNGS elevates diagnostic yield by 30% to 40%.^[24]^ These evidence-based recommendations underscore its role in high-stakes clinical scenarios while emphasizing context-dependent application.

## 4. Conclusion

A patient with septic cardiomyopathy induced by severe CAP-AB was treated with early VA-ECMO. Patients with CAP-induced septic cardiomyopathy may benefit from the introduction of VA-ECMO during the early stages. Further studies are required to evaluate the advantages and disadvantages of early VA-ECMO in patients with CAP-induced septic cardiomyopathy.

## Acknowledgments

We would like to express our gratitude to the patient for granting permission to use his clinical data in this study and for the publication of this research.

## Author contributions

**Conceptualization:** Yan-Na Jiao.

**Data curation:** Yan-Na Jiao.

**Formal analysis:** Yan-Na Jiao.

**Funding acquisition:** Jian-Biao Meng.

**Investigation:** Yan-Na Jiao.

**Methodology:** Yan-Na Jiao.

**Supervision:** Jian-Biao Meng.

**Writing – original draft:** Yan-Na Jiao.

**Writing – review & editing:** Jian-Biao Meng.

## Supplementary Material


