# Supplementary material for: Veno-arterial extracorporeal membrane oxygenation in a patient with septic cardiomyopathy induced by severe community-acquired pneumonia due to Acinetobacter baumannii: A case report
Source: Medicine (Baltimore). 2025 May 23;104(21):e42092. doi: 10.1097/MD.0000000000042092 (PMC12114006; doi:10.1097/MD.0000000000042092)
Supplement: Supplementary file 1 [file medi-104-e42092-s001.pdf]

Supplemental Materials

Supplemental table 1 Reference ranges of physiological indexes

**Physiological indexes**	**Reference ranges**
body temperature (□)	36.3-37.2
heart rate (beats/min)	60-100
respiratory rate (breaths/min)	12-20
blood pressure (mmHg)	90-140/60-90
pulse oxygen saturation (SpO_2) (%)	98-100 on room air
left ventricular ejection fraction (LVEF) (%)	53-76
tricuspid annular plane systolic excursion (TAPSE) (mm)	≥20
right ventricular global longitudinal strain (RVGLS) (%)	≥23
central venous pressure (CVP) (mmHg)	6-12
arterial blood pressure (ABP) (mmHg)	90-140/60-90
global end diastolic volume index (GEDVI) (ml/m^2)	680-800
cardiac index (CI) (ml/m^2/min)	3.5-5.0
systemic vascular resistance index (SVRI) (dyns/cm^5m^2)	1700-2400
extravascular lung water index (EVLWI) (ml/kg)	3-7

Supplemental Materials

Supplemental table 2 Reference ranges of laboratory indices

Laboratory indices	Reference ranges
white blood cell count (/ml)	4000-10000
hemoglobin (g/L)	130-175
platelet count (/ml)	125000-350000
C-reactive protein (hs-CRP) (mg/L)	0-6.00
pH	7.35-7.45
PaO_2 (mmHg)	80-100 on room air
PaO_2/ FiO_2 (mmHg)	>300 on room air
lactic acid (mmol/L)	0.5-1.6
procalcitonin (PCT) (ng/mL)	<0.065
high-sensitivity troponin T (hs-TNT) (ng/L)	<0.017
heart type-fatty acid binding protein (HFABP) (ng/mL)	<5.00
N-terminal pro B-type natriuretic peptide (NT-proBNP) (pg/mL)	<125
HCO_3^- (mmol/L)	21.0-28.0
O_2 saturation (%)	98-100 on room air
